# The role of B-Cell Lymphoma-3 (BCL-3) in enabling the hallmarks of cancer: implications for the treatment of colorectal carcinogenesis

**DOI:** 10.1093/carcin/bgaa003

**Published:** 2020-01-13

**Authors:** Danny N Legge, Adam C Chambers, Christopher T Parker, Penny Timms, Tracey J Collard, Ann C Williams

**Affiliations:** Colorectal Tumour Biology Group, School of Cellular and Molecular Medicine, Faculty of Life Sciences, Biomedical Sciences Building, University Walk, University of Bristol, Bristol, UK

## Abstract

With its identification as a proto-oncogene in chronic lymphocytic leukaemia and central role in regulating NF-κB signalling, it is perhaps not surprising that there have been an increasing number of studies in recent years investigating the role of BCL-3 (B-Cell Chronic Lymphocytic Leukaemia/Lymphoma-3) in a wide range of human cancers. Importantly, this work has begun to shed light on our mechanistic understanding of the function of BCL-3 in tumour promotion and progression. Here, we summarize the current understanding of BCL-3 function in relation to the characteristics or traits associated with tumourigenesis, termed ‘Hallmarks of Cancer’. With the focus on colorectal cancer, a major cause of cancer related mortality in the UK, we describe the evidence that potentially explains why increased BCL-3 expression is associated with poor prognosis in colorectal cancer. As well as promoting tumour cell proliferation, survival, invasion and metastasis, a key emerging function of this proto-oncogene is the regulation of the tumour response to inflammation. We suggest that BCL-3 represents an exciting new route for targeting the Hallmarks of Cancer; in particular by limiting the impact of the enabling hallmarks of tumour promoting inflammation and cell plasticity. As BCL-3 has been reported to promote the stem-like potential of cancer cells, we suggest that targeting BCL-3 could increase the tumour response to conventional treatment, reduce the chance of relapse and hence improve the prognosis for cancer patients.

## Teaser

In recent years there has been an increasing number of studies investigating the role of the atypical IκB protein BCL-3 in a wide range of human cancers; here we review its function in relation to the ‘Hallmarks of Cancer’.

## Introduction

Globally, cancer is one of the most common causes of mortality, with colorectal cancer the third most common malignancy and fourth highest cause of cancer death (1). Tumourigenesis is driven through acquisition of somatic mutations, epigenetic modifications (such as alterations in alternative splicing) and regulation by non-coding RNA molecules that function to regulate the expression of tumour suppressor and oncogenes. These changes lead to phenotypic alterations that mark the transformation of normal cells to cancer cells, known as the ‘Hallmarks of Cancer’, as originally defined by Hanahan and Weinberg (2). These Hallmarks are evading apoptosis, self-sufficiency in growth signals, insensitivity to anti-growth signals, limitless replicative potential, sustained angiogenesis and tissue invasion and metastasis. An updated version of the hallmarks describes a further two ‘emerging hallmarks’; deregulating cellular energetics and avoiding immune destruction, along with two ‘enabling characteristics’; genome instability and tumour promoting inflammation (3).

### NF-κB is a critical inflammatory signalling pathway

**Figure 1.**
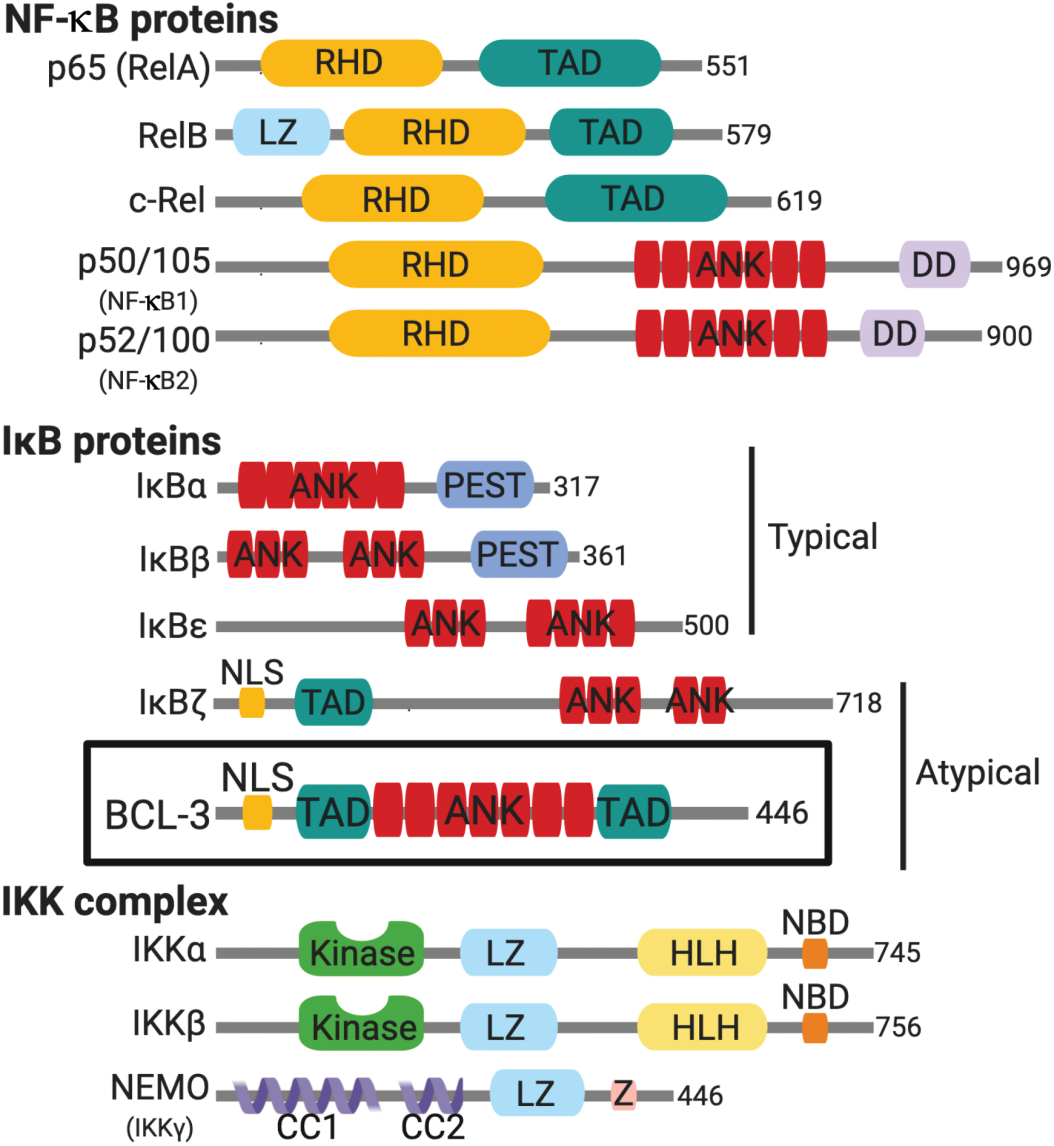
Principal components of NF-κB signalling. NF-κB signalling relies on three principal proteins: NF-κB proteins, IκB proteins and the IKK complex. The five members of the NF-κB family: p65 (RelA), RelB, c-Rel, p50/105 (NF-κB1) and p50/105 (NF-κB2) all share a N-terminal Rel homology domain that is responsible for DNA binding and dimerization. RelA, p65 and RelB also have a C-terminal transcriptional activation domain (TADs) which mediates interactions with co-factors but is not present in p50/100 or p52/105. The IκB proteins: IκBα, IκBβ, IκBε, BCL-3 and IκBζ are characterized by the presence of multiple ankyrin repeat domains (ANK = red). These domains assemble into elongated cylinders and associate with the DNA-binding domains of NF-κB dimers. Classically, IκB proteins sequester NF-κB dimers in the cytoplasm rendering them transcriptionally inactive. Atypical IκB proteins including BCL-3, the focus of this review, contain additional nuclear localization signal and transactivation domains. Unlike typical IκB proteins, these atypical nuclear IκB proteins can both inhibit and activate NF-κB target gene expression. The IKK complex contains two kinase subunits IKKα and Inhibitor of Nuclear Factor Kappa B Kinase Subunit Beta and a regulatory subunit NEMO (NF-κB essential modifier) or IKKγ. The total number of amino acids in each protein is indicated on the right-hand side. Leucine-zipper-like motif (LZ), death domain (DD), domain rich in proline (P), glutamate (E), serine (S) and threonine (T) (PEST), Kinase domain (Kinase), helix–loop–helix domain (HLH), NEMO-binding domain (NBD), coiled-coil domain (CC) and zinc-finger domain (Z). Figure created with BioRender.com.

The NF-κB family of transcription factors is quickly activated following numerous stress stimuli including infection (4), ionizing radiation (5), chemical/physical stress and pro-inflammatory signals—including those related to cancer (6, 7). NF-κB signalling relies on three principal protein families: NF-κB proteins, inhibitor of kappa B (IκB) proteins and the IKK complex (Figure 1). NF-κB proteins are a group of structurally related subunits: RelA (p65), RelB, c-Rel, NF-κB1 (p105/p50) and NF-κB2 (p100/p52) (8, 9). All NF-κB subunits contain an N-terminal Rel homology domain, which mediates DNA binding, dimerization of subunits and inhibitory protein binding (10). P65, RelB and c-Rel possess a transactivation domain which enables transcriptional activation following stimulus. Dimerization of NF-κB subunits into homo- or heterodimers follows activation through either the canonical or non-canonical pathways, although this is perhaps an oversimplification as up to 15 different NF-κB family complexes can be formed (11, 12). The canonical and non-canonical signalling pathways have been the subject of many elegant reviews and are not covered here.

The term ‘atypical NF-κB pathway’ involves NF-κB dimers and their associated activation that do not fall under the umbrella of the canonical or non-canonical pathways: included in this atypical calssification are the p50/p52 homodimers and their associated co-regulatory protein, B-Cell Lymphoma-3 (BCL-3), which will be the focus of this review. This pathway is a critical component of the broader NF-κB response and plays a role in fine-tuning the canonical and non-canonical responses to an inflammatory stimulus. However, it is important to point out that BCL-3 may elicit functional effects through all branches of NF-κB signalling due to its various nuclear and cytoplasmic roles, as explored throughout this review.

## BCL-3

The BCL-3 gene was first found in chronic lymphocytic leukaemia after sequencing of recurring t(14;19)(q32.3;q13.1) translocations which result in transcriptional upregulation of *BCL3* (13). This revealed a protein with seven ankyrin repeat domains, a proline-rich N terminal domain and a serine- and proline-rich C-terminal domain with a total molecular weight of around 47 kDa (14). Studies in normal blood cells showed increased expression of BCL-3 following mitogenic stimulation and also linked the structure of BCL-3 to those of known cell cycle regulators, prompting the authors to label it as a candidate proto-oncogene (14). Shortly after its discovery, a flurry of papers from multiple groups linked BCL-3 to the NF-κB signalling pathway (15–18) and BCL-3 was subsequently classified as an atypical member of the IκB family due to its contrasting roles in NF-κB-mediated transcription.

### BCL-3 as a regulator of an atypical NF-κB signalling pathway

**Figure 2.**
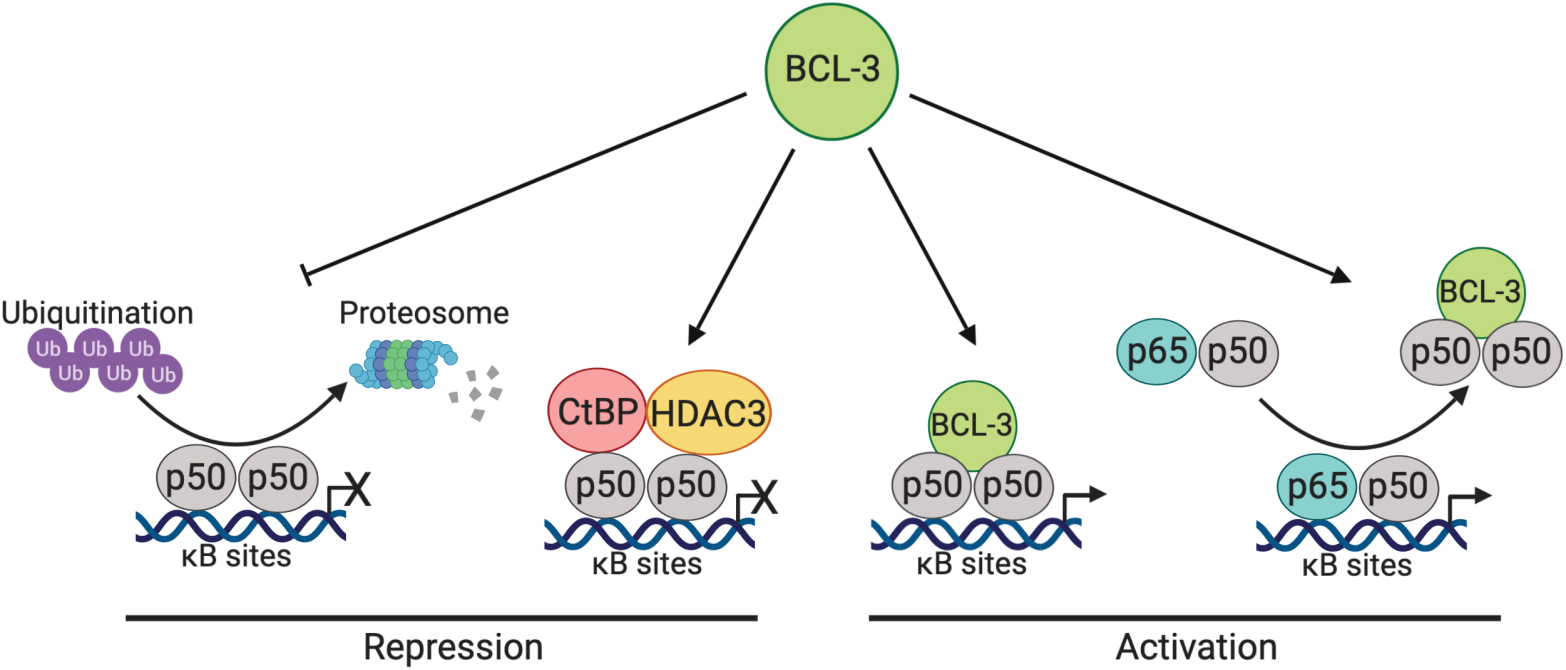
BCL-3 regulation of the atypical NF-κB signalling pathway. The atypical pathway is defined as the activation of the NF-κB p50 and p52 homodimers. BCL-3 acts as a regulator of the atypical NF-κB pathway by binding to processed p50 and p52 homodimers to repress or activate a subset of NF-κB regulated genes. In terms of transcriptional regulation, BCL-3 stabilizes repressive p50 homodimers bound to κB sites by inhibiting ubiquitination and subsequent proteolytic degradation of p50 homodimers. Additionally, BCL-3 can also recruit co-repressors CtBP and HDAC3 to repress transcription of NF-κB target genes. Conversely, BCL-3 directly activates transcription of NF-κB target genes by associating with p50 and p52 homodimers, providing these homodimers with two transactivation domains that they otherwise lack. BCL-3 can also remove repressive p50 homodimers situated at κB sites on DNA, allowing NF-κB heterodimers (p65/p50) associated with canonical signalling to activate transcription at these sites. Whilst BCL-3 is known to directly interact with p52 homodimers, the mechanism by which BCL-3 regulates p52 homodimer activation remains to be elucidated however it is speculated to follow a similar mechanism to p50 homodimers. Figure created with BioRender.com.

Functionally, BCL-3 differs from the other IκB proteins (IκB-α, IκB-β and IκB-ε) which bind to NF-κB proteins in the cytoplasm, inhibiting their nuclear translocation and subsequent transcriptional activity. In both non-tumour and tumour cells, BCL-3 binds to processed p50 and p52 homodimers to activate or repress a subset of NF-κB regulated genes (Figure 2). P50 and p52 are thought to form strong homodimers with high affinity compared with other homodimer species (19). These homodimers bind to the majority of κB sites at gene promoter regions and binding is known to occur at multiple sites within the same promoter (20). In contrast to other NF-κB subunits, p50 and p52 lack transactivation domains and subsequently require co-factors to induce activation of transcription (21). First thought to inhibit nuclear translocation of p50 homodimers (22), binding of BCL-3 to p50 homodimers was later shown to unmask the nuclear localization signal of p50 allowing translocation of the complex into the nucleus (23, 24).

### Transactivator or transrepressor

There have been many studies investigating the role of BCL-3 in the regulation of NF-κB target genes. Interestingly, BCL-3 has been demonstrated to both activate and repress transcription of NF-κB targets in a context-dependent manner with respect to the promoter, cell type or stimuli analysed. In terms of transcriptional repression, early work from Kerr *et al.* described inhibition of NF-κB reporter activity in the presence of BCL-3 in *Drosophila* S2 cells and Jurkat T-cells (18). In support of this, using cells from Bcl3^−/−^ mice it was demonstrated that Bcl-3 could enhance repression of NF-κB target genes. This was shown to be through inhibition of ubiquitination and subsequent proteolytic degradation of p50 homodimers bound to κB sites at promoters of certain genes; thus, extending the half-life of repressive p50 homodimers (25, 26). In addition, BCL-3 can recruit co-repressors CtBP and HDAC3 to repress transcription of NF-κB target genes in transformed keratinocytes (27). Therefore, BCL-3 can repress transcription by stabilizing repressive p50 homodimers or via recruitment of co-repressors.

Conversely, BCL-3 may activate transcription using a number of different mechanisms. Franzoso *et al.* demonstrated an interaction between BCL-3 and p50 and observed an increase in NF-κB reporter activity through BCL-3-mediated reversal of p50 homodimer-induced repression in transfected NTERA2 cells (17). A further mechanism of transcriptional activation by BCL-3 is the direct removal of repressive p50 homodimers already situated at κB sites on DNA, allowing NF-κB subunits associated with canonical signalling to bind and activate transcription at these sites (17, 28). As an alternative to reversing repression at κB sites, BCL-3 may also directly transactivate genes in multiple cell types when associated with p50 or p52 homodimers; owing to BCL-3 possessing both N- and C-terminal transactivation domains (14, 29, 30).

### Interaction of BCL-3 with co-regulatory proteins

BCL-3 can recruit other co-regulators to influence gene expression, as reported by Dechend *et al.* who discovered interactions between BCL-3 and Pirin, Tip60, Jab1 and Bard1. Of these interactors, Tip60 and Jab1 were found to enhance transcription of the P-selectin promoter in *Drosophila* SL2 cells when co-transfected with Bcl-3 and p50 (31). This adds a further dimension to the regulation of transcription by BCL-3, with some co-regulators resulting in repression of target genes and others in transactivation. Further protein–protein interactions have been documented such as with Hsp70, HDAC1 and 3, CtBP1 and CtBP2 (27, 32) and more recently β-catenin in colorectal cancer cells (33).

Acetylation is known to play an important role in NF-κB family subunits (34), although it is unknown if the bridging role that BCL-3 plays with proteins such as Tip60 and HDAC1/3 facilitates acetylation of p50 or p52 homodimers. Evidence exists to show that p50 can be acetylated and that this acetylation results in enhanced binding to κB sites in T cells (35), it is interesting to speculate that Tip60 and HDAC1/3 interactions could be influential in determining homodimer transactivation activity in the nucleus.

**Figure 3.**
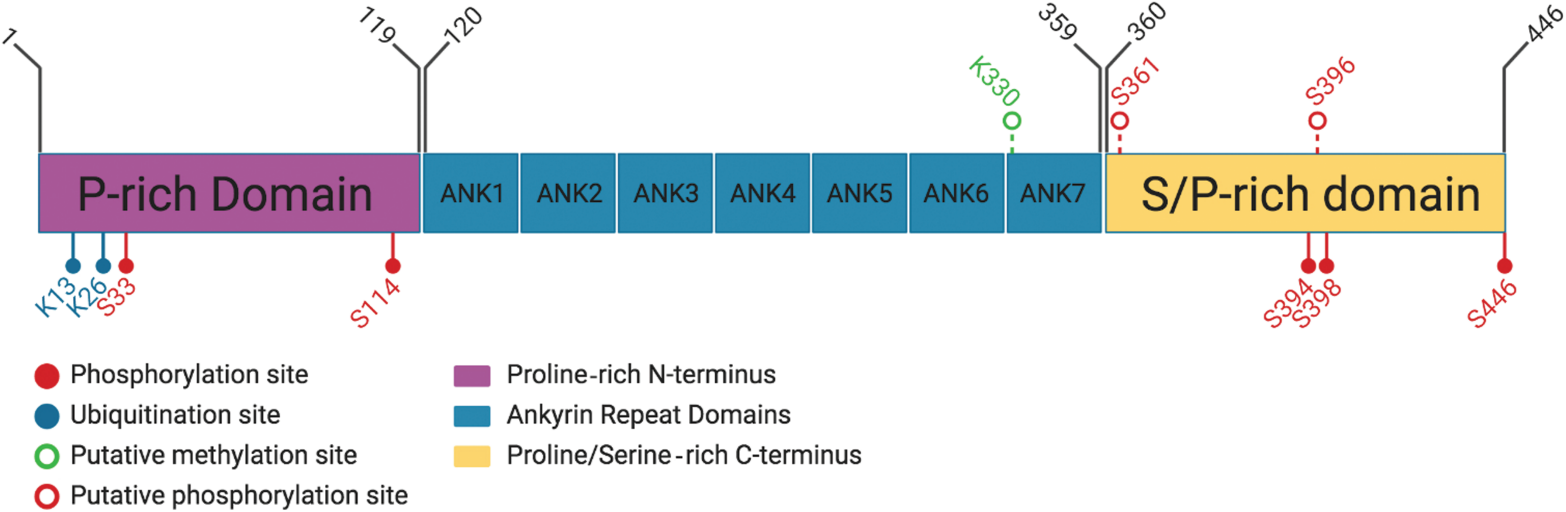
Gene map of BCL-3 highlighting important sites of post-translational modification. BCL-3 is 446 amino acids in length with a proline-rich N-terminus, an ankyrin repeat region consisting of seven ankyrin repeats and a proline- and serine-rich C-terminus. In the C-terminus, serine residues S361 and S396 are GSK3β targets and result in protein degradation. S33, S114 and S446 are phosphorylated by AKT, ERK2 and IKK1/2, respectively, leading to stabilization of BCL-3. Lysine residues K13 and K26 undergo ubiquitination with either stabilizing K48 or degradative K63 ubiquitin chains. Other putative PTM sites in BCL-3 are lysine residue K330 that is thought to be monomethylated in resting cells and two potential sites for ATM phosphorylation, S361 and S396, both of which are followed by the typical glycine residue found in ATM target sites. Figure created with BioRender.com.

Although early work on BCL-3 focussed on its transcriptional regulation in relation to its interaction with NF-κB homodimeric subunits, more recent work has centred on how post-translational modifications affect these interactions and downstream transcriptional regulation (Figure 3 summarizes the key modifications of BCL-3). BCL-3 is extensively phosphorylated at its C-terminus, which regulates p52 (36) and p50 (24, 37) homodimer binding. In NIH3T3 cells, Viatour *et al.* demonstrated phosphorylation of BCL-3 at residues S394 and S398 by glycogen synthase kinase 3 promoted proteasomal degradation of BCL-3 (38). A recent mass spectrometry study in HEK293T cells has revealed 27 phosphorylation sites on BCL-3 (39). Subsequent characterization revealed phosphorylation at S33 by Akt shifts polyubiquitination from K48 to K63, resulting in resistance to degradation, translocation to the nucleus and activation of p52 homodimers (39). S446 phosphorylation by IKK1/2 and S114 phosphorylation by Erk2 both stabilized the BCL3:p52 complex on DNA to enhance transcriptional activation (39). Therefore, whether BCL-3 acts as a co-activator or co-repressor may be determined by the combination of post-translational modifications—brought about by the particular cell type or stimuli—that alter the function and/or localization of BCL-3.

In summary, BCL-3 has multiple functions ranging from activation of p50/p52 subunits in the cytoplasm, their nuclear import and modulation of homodimer transactivation through facilitating binding of different transcriptional co-regulators. It is also suggested that BCL-3 has contrasting functions depending on the pathological model used; in particular there is a significant reversal of function in inflammation models (40) which could be explained by the availability of co-factors and/or post-translational modification of chromatin surrounding NF-κB dimers (41).

## BCL-3 and cancer

Given its identification as a proto-oncogene in chronic lymphocytic leukaemia and central role in regulating NF-κB signalling, it is perhaps not surprising that there have been an increasing number of studies in recent years investigating the role of BCL-3 in a variety of human solid cancers (42). There are multiple post-translational modifications that affect the function or localization of BCL-3, as discussed above, and may promote the oncogenic potential of BCL-3.

The clinical utility of BCL-3 as a marker of prognosis is highlighted by a growing number of clinical studies: increased protein and mRNA expression has been associated with adverse clinicopathological characteristics in hepatocellular carcinoma patients (43). Increased BCL-3 expression was also correlated with adverse prognostic features and reduced survival rate in cervical cancer (44). Niu *et al.* suggest that BCL3 is a promising molecular biomarker of paediatric acute myeloid leukaemia with unfavourable prognosis (45), and the use of BCL-3 expression levels has also been proposed to predict response to alkylating agents in glioma (46). Interestingly, BCL-3 is part of a four gene signature suggested for use in determining the prognosis of patients with clear-cell renal-cell carcinoma (47). These and other studies have begun to shed light on our mechanistic understanding of the function of BCL-3 in tumour promotion and progression, including the role BCL-3 plays in a number of the Hallmarks of Cancer (3), as discussed below.

### Sustaining proliferative signalling

Cancer cells can disregard the homeostatic cues that govern normal cells ability to grow and, importantly, stop growing. Cancers acquire the ability for self-sustaining proliferation through a number of different means, including dysregulation of cell cycle, increased growth factor production, stimulation of cells within the microenvironment (resulting in increased pro-proliferative paracrine signalling) and the deregulation of growth factor receptors leading to hypersensitivity to growth factors (3, 48). The effect of BCL-3 on tumour growth/proliferation has been observed in different tumour types. In colorectal cancer cells, BCL-3 induces the post-translational stabilization of c-MYC (mediated by ERK1/2) increasing tumour xenograft size (49), while BCL-3 overexpression was shown to induce cell cycle progression, mediated by Cyclin D1 in hepatocellular carcinoma (43, 50), malignant melanoma (51) and breast cancer (52). The converse was also true, as repression of Cyclin D1 by p53 was shown to be dependent on BCL-3 suppression in H1229 lung cancer and U2-01 sarcoma cell lines (53). Furthermore, proliferation of skin cancer cells was abrogated when nuclear translocation of BCL-3 was blocked by its upstream regulator, CYLD (54).

### Activating invasion and metastasis

Invasion into surrounding tissues is a defining feature of malignant cells followed by metastasis to distant organ sites, which is often the fatal event of solid malignancy (3). In breast cancer models it has been demonstrated that BCL-3 drives metastasis of tumour cells (55, 56): In HER2-positive breast tumour cells, BCL-3 knockout (KO) resulted in an 80% reduction of metastatic burden in mice following tail-vein injection of tumour cells (55). While in the MMTV-PyMT mouse model of mammary adenocarcinoma, Bcl-3 suppression using Dox-inducible shRNA resulted in reduction of lung metastases, through targeting Smad3 stability in the transforming growth factor-β signalling pathway (56). Additionally, a clinical study examined immunohistochemistry from paired normal and tumour tissue in hepatocellular carcinoma and discovered BCL-3 expression resulted in advanced Tumour, Node, Metastasis stage; this was shown to have contributed to the poorer prognosis observed in these patients (43).

### Evasion of apoptosis

BCL-3 regulates cellular apoptosis in a variety of models. For example, data have shown BCL-3 regulates apoptosis in colorectal and cervical tumour cell lines following UV-radiation (57) and protects breast carcinoma cells from undergoing apoptosis following UV-radiation (58). BCL-3 is thought to inhibit proteins such as Smac/Diabolo and p53 through upregulation of HDM2 (59–62). However, even in p53-null backgrounds BCL-3 suppression was able to initiate apoptosis through targeting the expression of DNA-PKcs (59). BCL-3 is a potent survival factor in colorectal cancer (63, 64), and activates the pro-survival AKT/PKB pathway (64). In hepatocellular carcinomas, BCL-3 is frequently overexpressed in tumour tissue compared with normal tissue, in conjunction with p50 and p52 NF-κB subunits (65). However, recently published data using a hepatocyte-specific BCL-3 overexpression mouse model revealed that BCL-3 expression promoted hepatocyte death following an inflammatory insult. As a result, these mice developed fewer hepatocellular carcinomas (66). This study also highlighted that BCL-3 overexpression reduced the influx of certain populations of immune cells into the liver (CD8^+^ T cells, B cells and leucocytes), protecting against induced inflammation. It may be that hepatocellular carcinomas are particularly sensitive to alterations in canonical NF-κB signalling and that repression of canonical NF-κB occurs when BCL-3 is overexpressed in combination with atypical NF-κB homodimers, leading to abrogation of apoptosis in this context (26). Interestingly, a recent study by Zou *et al.* described BCL-3-mediated increase in checkpoint marker PD-L1 expression, enhancing proliferation of ovarian cancer cells (67). PD-L1 is known to inhibit tumour infiltrating lymphocytes (68); therefore, it is tempting to speculate that BCL-3 could play a similar role in protecting against tumour cell apoptosis via regulation of tumour infiltrating lymphocytes in both hepatocellular carcinoma and ovarian cancer.

### The role of BCL-3 in inflammation and immunity

NF-κB signalling is recognized to have contrasting function depending on the biological context (69), consistent with the role of BCL-3 in cancer compared with its role in non-malignant, inflammatory models. Interestingly, the presence of BCL-3 appears to attenuate inflammation in non-malignant models. BCL-3 KO mice have defects in lymphoid organs, including aberrant development of Peyer’s patches, which would affect intestinal immune surveillance and response to certain pathogens (70).

Effects of BCL-3 KO have been studied in a variety of different inflammatory models, including pancreatitis, colitis, dermatitis and rheumatoid arthritis. In a mouse model of acute pancreatitis, where pancreatic inflammation is stimulated using cerulin or sodium taurocholate, results showed that Bcl-3^−/−^ mice had increased levels of oedema and necrosis in their pancreata (71). The observation that Bcl-3 KO increases the severity of inflammation has been corroborated in an inflammatory bowel disease model (Crohn’s disease and ulcerative colitis) (40). In a contact hypersensitivity model of atopic dermatitis, Bcl-3^−/−^ mice had worsened inflammation following topical oxalazone treatment, which appeared to be mediated through increased cytokine production (72). Earlier evidence for this mechanism came from Carmody *et al.* via examining the expression of pro-inflammatory cytokines in murine immune cells (macrophages and dendritic cells) devoid of BCL-3 (26). BCL-3 acted to repress canonical NF-κB transcription of target cytokines (such as TNF-α, CXCL1, CXCL2, IL-1β and IL-10), leading to significantly increased expression following inflammatory stimuli in the Bcl-3 KO cells, corroborating previous data (73). This study was particularly interesting as it demonstrated that not all cytokines responded in the same way to Bcl-3 KO. For cytokines that showed an early spike in transcription following lipopolysaccharide, Bcl-3 KO had a significant impact on their production, while cytokines such as IL-6 showed a slower increase in transcription that was unchanged in Bcl-3^−/−^ cells compared with Bcl-3 wild-type controls. These data suggest that Bcl-3 KO affects different aspects (early and late) of the NF-κB-driven response to inflammation. Interestingly, Inhibitor of Nuclear Factor Kappa B Kinase Subunit Beta KO in intestinal epithelial cells worsens the histological severity of colonic inflammation and led to the animals losing greater amounts of weight (74). If Bcl-3 KO leads to increased binding of canonical NF-κB dimers (26), then it is unclear why the phenotype observed with Bcl-3 KO is similar to the phenotype observed when canonical NF-κB has been inactivated by Inhibitor of Nuclear Factor Kappa B Kinase Subunit Beta deletion. It may be that long-term KO and transient knockdown have different effects on the canonical (transiently activated) and non-canonical pathway (sustained activation). Additionally, the various models and cell types used in these studies may account for some of the context-dependent function of Bcl-3.

As a further illustration of the potential context-dependent function of BCL-3, another recent study showed that Bcl-3 overexpression, specifically in CD4^+^ T cells (including T regulatory cells), results in a pancolitis of the large bowel of mice (75). This cell-type specific effect was corroborated by work in CD4^+^ cells using a rheumatoid arthritis model, which showed overexpression of Bcl-3 in these cells was implicated in the pathogenesis of rheumatoid arthritis (76). Interestingly, Bcl-3^−/−^ T cells failed to induce colitis when transferred into Rag1^−/−^ mice (77), suggesting an inability of Bcl-3^−/−^ T cells to respond to the microbiota-derived and antigen-specific signals that drive colitis in this model.

Following induction of inflammation there is a concomitant induction of BCL-3 in a variety of tissue types. Induction of BCL-3 in response to canonical NF-κB signalling was first observed by Brasier *et al.* in a hepatocellular carcinoma background (78). More recent work has shown the importance of the alternative NF-κB pathway in regulation of BCL-3 transcription in colorectal cancer (63). This is likely to represent a feedback loop, in place to regulate pathways such as NF-κB and modulate their function following a stimulus. Evidence for this comes from a number of sources; in respiratory syncytial virus infection of airway epithelial cells, BCL-3 is initially upregulated leading to inhibition of NF-κB and STAT transcription by acting as a bridging factor to HDAC1, which is transcriptionally repressive (32). Furthermore, BCL-3 represses STAT3 regulating proteins (79); repression of STAP2 by BCL-3 and p50 homodimers in conjunction with CtBP on the STAP2 promoter can diminish STAP2-dependent BRK kinase signalling that phosphorylates and activates STAT3 (80). BCL-3 is upregulated in tissue from patients with inflammatory bowel disease (75), although further data from inflammatory bowel disease patients have shown that CpG sites in the *BCL3* gene are commonly methylated and therefore repressed in B cells isolated from diseased tissue (81). Bcl-3^−/−^ granulocytes, isolated following pulmonary transplant in mice, had higher levels of apoptosis as measured by annexin V (82). This links to other data showing Bcl-3 KO cells in mice colons displayed increased caspase-3 cleavage following dextran-sodium sulphate-induced colitis (40), suggesting these cells were undergoing more apoptosis. Despite these data showing the role of BCL-3 as both a pro-inflammatory and an anti-inflammatory mediator in non-cancer tissue, it does still appear that BCL-3 has similar control over regulation of apoptosis in non-malignant tissue compared with the role of BCL-3 in cancers.

In summary, BCL-3 has alternative roles in inflammation depending on the cell type it is expressed in and whether all tissues have lost or gained expression. It is suggested that KO of Bcl-3 reduces the ability of cells to upregulate survival programmes following an inflammatory insult, leading to concomitant rise in apoptosis. This results in a worse grade of histological inflammation and tissue damage, thereby propagating inflammation. This indicates that it may be the pro-survival role of BCL-3 following stress or insult which is critical to its function.

## BCL-3 in colorectal cancer

Importantly, BCL-3 has been shown to play multiple roles in the promotion of colorectal cancer. It was discovered by Puvvada *et al.* that BCL-3 nuclear expression in primary tumours was negatively associated with survival. This was calculated through analysis of staining and microarray data gathered from tumour specimens of 23 patients that had undergone colorectal cancer resection (83). Strong nuclear BCL-3 staining was also reported in 33% of 270 tumour samples, and was suggested to be an important diagnostic determinant (84). These findings were recently corroborated by Legge *et al.* who (using publically available datasets) also reported that BCL-3 expression is associated with poor prognosis in colorectal cancer (33). Various mechanisms of BCL-3-mediated tumour promotion have been observed in colorectal cancer. One of these is suggested to be through regulation of Cyclin D1 in SW480 cells, after it was observed that expression of BCL-3 and Cyclin D1 was reduced following treatment with COX-2 inhibitor NS398 (85). Work from our group has shown that BCL-3 promotes colorectal tumour cell survival and growth *in vivo*, through activation of the AKT pathway via phosphoinositide 3-kinase and mammalian target of rapamycin (mTOR) signalling (64). Moreover, BCL-3 also stabilizes c-MYC—one of the key oncogenes in early stage colorectal tumourigenesis following adenomatous polyposis coli inactivation—via extracellular signal-regulated kinase signalling (49).

Conversely, a recent paper has suggested Bcl-3 may play a protective role in colitis-associated colorectal cancers. This observation was made in the mouse model of colitis associated colorectal cancers, where tumours are induced through the addition of azoxymethane/dextran-sodium sulphate. Tang *et al*. demonstrated that mice with conditional deletion of Bcl-3 in the intestine developed significantly more polyps than wild-type controls, though interestingly polyps from both animals were the same size and staining of proliferation markers were comparable, indicating Bcl-3 loss was promoting tumour initiating capacity. This was suggested to be mediated through TNF-α, as tumour burden was not increased in Bcl-3/TNF-α double KO mice (86). Interestingly, earlier work by O’Carroll *et al.* revealed increased proliferation of intestinal epithelium in Bcl-3^−/−^ mice compared with controls, showing that this preserved intestinal tissue architecture, protecting against colitis (40). It is tempting to speculate that the increased proliferation in Bcl-3^−/−^ intestines following dextran-sodium sulphate administration may contribute to tumour development observed in the Tang *et al.* study. It is important to remember that in the O’Carroll study, germline Bcl-3 KO will affect the immune cell compartment which will play a key role in colitis associated colorectal cancers, as discussed in the previous section.

### BCL-3 and colorectal cancer stem cells

Cancers are thought to arise from stem (or stem-like) cells that gain a competitive advantage over their neighbours. With time this clonal expansion of mutant cells will eventually give rise to a tumour (87). The cancer stem cell hypothesis states there are cells within tumours capable of self-renewal, in addition to producing other heterogeneous, differentiated cell types that constitute the tumour mass (88). In the colon, deregulation of Wnt signalling is recognized as the key initiating event in colorectal cancer; activation of Wnt/β-catenin signalling, most commonly through inactivating mutations in adenomatous polyposis coli, leads to the formation of benign polyps, the first stage in the adenoma to carcinoma sequence (89). Important studies in stem cell biology have begun to identify the mechanisms underpinning the expansion of the adenomatous polyposis coli mutant stem cells that contribute to the earliest stages of colorectal tumour development (90), which may represent novel targets for therapeutic intervention.

Previous data have highlighted the importance of the interplay between NF-κB signalling and the Wnt/β-catenin pathway in colorectal carcinogenesis; in particular that non-stem cells engineered to exhibit high levels of Wnt and NF-κB signalling can de-differentiate, initiating tumours in mice (91). Recently we have shown that BCL-3 is an important co-activator of β-catenin/T-cell factor-mediated transcriptional activity in colorectal cancer cells, increasing expression of Wnt-regulated intestinal stem cell genes. BCL-3 was demonstrated to bind to β-catenin; RNAi-mediated BCL-3 suppression reduced β-catenin/T-cell factor-dependent transcription and the expression of intestinal stem cell genes and Wnt targets LGR5 and ASCL2, both widely accepted colorectal cancer stem cell markers (92–96). Furthermore, we showed that BCL-3 promotes the stem cell phenotype in colorectal cancer cells by increasing colorectal spheroid and tumoursphere formation in 3D culture conditions, indicating BCL-3 may promote tumour initiation *in vivo* and may be an effective target for reducing cancer stem cell plasticity (33). Results from our study complement findings by Chen *et al.* who suggest BCL-3 promotes stem-like activity and contributes to the maintenance of naive pluripotency in mouse embryonic stem cells (97). Due to its role in regulating both β-catenin and NF-κB signalling, it is interesting to speculate that BCL-3 may enhance the de-differentiation of non-stem cells (91), therefore aiding reconstitution of the tumour when the cancer stem cells have been deleted (94).

## Summary

In conclusion, BCL-3 may represent an exciting new route for targeting the Hallmarks of Cancer; in particular by limiting the impact of the enabling hallmarks of tumour promoting inflammation and cell plasticity. Excitingly, targeting stem cell plasticity offers the possibility of overcoming some of the limitations of directly targeting cancer stem cell highlighted in recent studies (94). We suggest that targeting BCL-3 function (through suppressing the stem-like potential of cancer cells) would increase the tumour response to conventional treatment, reduce the chance of relapse and hence improve the prognosis for colorectal cancer patients. Given the emerging role of BCL-3 in a number of different cancers, we are hopeful that the new class of BCL-3 inhibitors currently under development will prove effective in a wide range of human cancers.

## Funding

This work was supported by PhD studentships from Bowel & Cancer Research PhD studentship (D.N.L., P.T.), an MRC clinical research training fellowship (MR/N001494/1 to A.C.C.), a Wellcome Trust Four Year PhD Programme in Dynamic Molecular Cell Biology (203988/Z/16Z to C.P.), an MRC Research grant (MR/R017247/1 to A.C.W., T.J.C.) and by the John James Bristol Foundation.

*Conflict of Interest Statement*: The authors have declared no conflicts of interest.

## Abbreviations

BCL-3: B-Cell Lymphoma-3
IκB: inhibitor of kappa B
